# Integrating genetic algorithms and language models for enhanced enzyme design

**DOI:** 10.1093/bib/bbae675

**Published:** 2025-01-08

**Authors:** Yves Gaetan Nana Teukam, Federico Zipoli, Teodoro Laino, Emanuele Criscuolo, Francesca Grisoni, Matteo Manica

**Affiliations:** IBM Research Europe, Säumerstrasse 4, CH-8803 Rüschlikon, Switzerland; Institute for Complex Molecular Systems and Department of Biomedical Engineering, Eindhoven University of Technology, 5612 AZ Eindhoven, the Netherlands; IBM Research Europe, Säumerstrasse 4, CH-8803 Rüschlikon, Switzerland; National Center for Competence in Research-Catalysis (NCCR-Catalysis), Switzerland; IBM Research Europe, Säumerstrasse 4, CH-8803 Rüschlikon, Switzerland; National Center for Competence in Research-Catalysis (NCCR-Catalysis), Switzerland; Institute for Complex Molecular Systems and Department of Biomedical Engineering, Eindhoven University of Technology, 5612 AZ Eindhoven, the Netherlands; Institute for Complex Molecular Systems and Department of Biomedical Engineering, Eindhoven University of Technology, 5612 AZ Eindhoven, the Netherlands; Centre for Living Technologies, Alliance TU/e, WUR, UU, UMC Utrecht, Utrecht, the Netherlands; IBM Research Europe, Säumerstrasse 4, CH-8803 Rüschlikon, Switzerland

**Keywords:** enzyme optimization, large language models, genetic algorithms, biocatalysis, computational protein design

## Abstract

Enzymes are molecular machines optimized by nature to allow otherwise impossible chemical processes to occur. Their design is a challenging task due to the complexity of the protein space and the intricate relationships between sequence, structure, and function. Recently, large language models (LLMs) have emerged as powerful tools for modeling and analyzing biological sequences, but their application to protein design is limited by the high cardinality of the protein space. This study introduces a framework that combines LLMs with genetic algorithms (GAs) to optimize enzymes. LLMs are trained on a large dataset of protein sequences to learn relationships between amino acid residues linked to structure and function. This knowledge is then leveraged by GAs to efficiently search for sequences with improved catalytic performance. We focused on two optimization tasks: improving the feasibility of biochemical reactions and increasing their turnover rate. Systematic evaluations on 105 biocatalytic reactions demonstrated that the LLM–GA framework generated mutants outperforming the wild-type enzymes in terms of feasibility in 90% of the instances. Further in-depth evaluation of seven reactions reveals the power of this methodology to make “the best of both worlds” and create mutants with structural features and flexibility comparable with the wild types. Our approach advances the state-of-the-art computational design of biocatalysts, ultimately opening opportunities for more sustainable chemical processes.

## Introduction

Computational models play a key role in enhancing the understanding and manipulation of complex biological systems [1–3]. Large language models (LLMs)—part of this broad set of computational tools—have shown unprecedented abilities in processing and generating text that closely mimics human writing [4, 5], and have permeated the chemical and biological sciences. Applying these models to biological sequences, especially proteins, has propelled the development of innovative methodologies for design and analysis [6–10]. By leveraging protein sequence databases, LLMs offer unprecedented insights into protein function and interactions, revolutionizing our understanding of biological systems [11].

Despite the widespread application of language modeling in protein optimization [12], there remains a critical gap: the complexity of optimizing protein sequences in such a high-cardinality space [13]. Our study aims to bridge this gap by integrating LLMs with dynamic optimization techniques, specifically through genetic algorithms (GAs) [14]. GAs have been applied to a broad range of complex optimization problems [15], spanning from drug discovery [16, 17] to material science [18]. Unlike traditional gradient-based optimization methods, GAs have a lower propensity to become trapped in local optima, making them particularly well suited for navigating the complex and multifaceted landscapes of protein sequences [19]. Their combination with LLMs has been used in the past for small molecules [20] but never scaled up to complex macro-molecules, such as enzymes.

**Figure 1:**
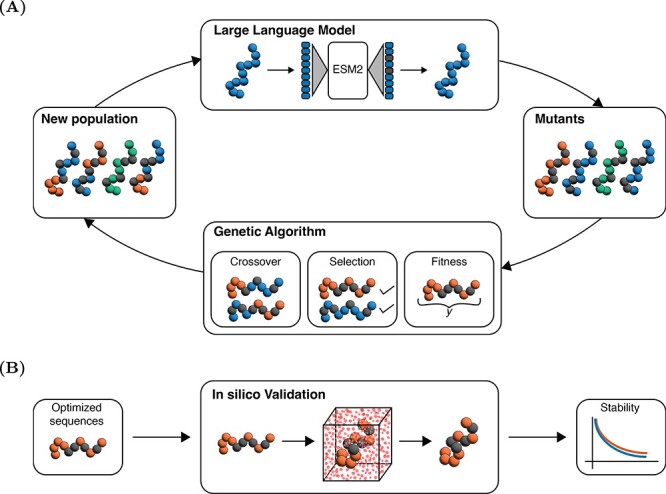
**Enzyme optimization framework**. (**A**) Our framework leverages an LLM to generate mutations of a protein sequence. The mutants are evaluated using a fitness function, and a subset is selected for crossover and the next iteration. The process is repeated until a stopping criterion is met, resulting in an optimized protein sequence. (**B**) The optimized sequence is then validated *in silico* through MD simulations.

In our study, we introduce a novel framework that combines the predictive power of LLMs with the adaptive mechanisms of GAs to accelerate enzyme design (see Fig. 1(A)). Within this framework, two steps occur: (a) LLMs direct the generation of a pool of mutants. (b) These mutants, represented as a varied collection of amino acid sequences, are then subject to the evolutionary processes simulated by GAs. The GA component performs a series of evolutionary steps to refine enzyme functionality, beginning with a phase where high fitness score sequences are selected for subsequent modifications. During the crossover phase, fragments from selected sequences is mixed to produce new variants. By iteratively cycling through this process, our framework progressively evolves enzyme sequences—in a synergy between LLMs prediction and GA evolution—with the ultimate goal of achieving enhanced catalytic performance.

Here, we test our methodology by applying it to a dataset comprising 105 biocatalytic reactions. We focused on two primary optimization objectives: (a) enhancing the likelihood of an enzyme (herein called feasibility) to catalyze specific reactions, and (b) optimizing the turnover number ($\text{Kcat}$), which is critical for enzyme efficiency. Our approach achieves 90% improvement in predicted feasibility across the test set and 100% in improving enzymes $\text{Kcat}$ values compared with the respective wild types. The robustness of our optimization framework was further corroborated by molecular dynamics (MD) simulations on a selected set of reactions. Taken together, our results highlight the ability of this integrated LLM–GA methodology to navigate the complex relationship between enzyme sequences and their catalytic performance.

## Materials and Methods

The methodology employed in this study leverages the synergistic combination of protein language models (LLMs) and GAs for enzyme sequence optimization. This approach aims to improve the catalytic efficiency and stability across a large protein sequence landscape. The use of LLMs for predictive analysis, paired with the evolutionary optimization capabilities of GAs, facilitates the targeted identification of beneficial mutations. Following optimization, MD simulations serve as a crucial validation step, verifying the structural integrity of the optimized enzymes.

### Dataset processing

Data for training and evaluating our fitness models were obtained from the BRENDA [21] and UniProtKB [22] databases. From BRENDA, 80 000 entries were extracted and matched with sequences in UniProtKB based on protein accession IDs. The dataset was then cleaned to remove entries with incomplete or ambiguous information and restructured to define one substrate, one product, and one associated enzyme per reaction, resulting in an expansion to approximately 180 000 entries. Standardization efforts included aligning with IUPAC nomenclature, eliminating entries not represented in PubChem [23], and refining the dataset to 119 000 samples. Samples were labeled “reported” to indicate verified substrate-product-enzyme associations, and were augmented with randomly generated substrate–product–enzyme pairings to test the optimization framework’s robustness. We collected a set of reactions ensuring a comprehensive representation across different biochemical processes by randomly selecting 50 reactions from each of the seven EC classes, totaling 350 reactions. The refinement to 105 reactions resulted from filtering based on the availability of matching enzyme structures in the Protein Data Bank (PDB) [24]. Specifically, we retained only those reactions where the enzyme’s sequence in the dataset had a 100% match with a sequence in the PDB. This selection criterion was crucial for accurately evaluating proteins through MD simulations. Our dataset encompasses biocatalyzed reactions from all seven main Enzyme Commission (EC) numbers, specifically containing a total of 3976 unique EC numbers. This extensive representation ensures a broad coverage of different biochemical processes, which is crucial for the generalizability of our findings across various enzyme classes and reaction types. Detailed analysis of the dataset reveals that transferases (EC 2.x.x.x) and oxidoreductases (EC 1.x.x.x) are the most represented classes, constituting 64.5% of the dataset. This is followed by hydrolases (EC 3.x.x.x), lyases (EC 4.x.x.x), ligases (EC 6.x.x.x), isomerases (EC 5.x.x.x), and translocases (EC 7.x.x.x), in descending order of frequency. This distribution reflects the general prevalence of these enzyme classes in biological processes [25].

### Mutation generation

In our enzyme optimization framework, we selected the ESM-2 model for mutation generation, based on its demonstrated excellence in protein sequence modeling [26, 27]. The initial step involved creating a varied population by masking amino acids within specific regions of the protein sequences to simulate potential mutation sites. ESM-2 then predicted the identities of these masked residues, effectuating the mutation process. For each mutated position, ESM-2 generated three potential mutations, selecting the most suitable amino acids based on its extensive knowledge of proteomics.

To validate the efficacy of ESM-2 within our framework, we conducted a comparative analysis between three mutation strategies: (a) a language model-based strategy utilizing ESM-2; (b) a transition matrix, named Smart, constructed from co-evolutionary data obtained through PSI-BLAST iterations (three iterations with 500 samples each) [28], reflecting amino acid conservation trends; and (c) a transition matrix, named Basic, that assigns uniform substitution probabilities across amino acids, without considering evolutionary or contextual data. This analysis covered 15 optimization cycles for 27 biochemical reactions, restricting each cycle to a population of 100 samples. The design incorporated variations in the maximum number of mutations per sequence (5, 10, and 15), tested across 30 unique random seeds to ensure methodological robustness. A pivotal part of our comparative analysis was employing the Wasserstein distance [29] to assess the efficiency of each mutation strategy throughout the optimization cycles.

In this analysis, we considered a reference distribution depicting the optimal case of algorithms runs converging in a single iteration. For each value of the maximum mutation parameter over all simulations ($n=7290$) grouped by mutation strategy, we computed the distribution of the iteration step corresponding to the first occurrence of the optimal score. We then calculated the Wasserstein distance between all simulations and the reference. This step allowed us to quantify the convergence speed of the different mutation strategies.

### Optimization with GAs

The optimization of enzyme sequences was performed using GAs [14]. Within the framework, the evaluation of the mutants is divided into two distinct models: the feasibility model and the $\text{Kcat}$ model. The feasibility model, implemented via a Random Forest (RF) algorithm with 100 decision trees [30], assesses the likelihood of a sequence to catalyze a reaction. The RF model is trained on a dataset comprising actual biocatalytic interactions (denoted “reported”) and “random” associations generated by randomly pairing substrate, product, and enzyme. The training data have been constructed to include elements from all EC classes. Training data for the model are numerical embeddings of substrates, products, and enzymes, reflecting their molecular structures and properties. Embeddings for substrate and product are derived from the ChemBERTa model [31], while enzyme embeddings are obtained from ESM-2. The output is a *feasibility score* ($F_{s}$) that quantifies the likelihood of a sequence facilitating successful catalysis. The RF model consists of 100 decision trees, each trained on a bootstrap sample of the training data using random subsets of features. During training, the Gini impurity criterion is used to select the optimal split at each node. The model is trained using five-fold cross-validation, where the dataset is split into five equal folds. In each iteration, four folds are used for training and the remaining fold for validation.

Conversely, the $\text{Kcat}$ model employs an XGBoost algorithm [32] for predicting the catalytic efficiency, characterized by the turnover number ($\text{Kcat}$). Prior to training, a logarithm transformation is applied to the dataset to improve the linearity between variables and reduce the impact of outliers. The model also utilizes embeddings for substrates and enzymes, extracted using the same models mentioned in the feasibility section. Following the methodology of Kroll *et al*. [33], the XGBoost parameters are learning rate $= 0.09$, max delta step $= 1.19$, min child weight $= 2.82$, reg alpha $= 1.94$, and reg lambda $= 4.95$. The R-squared ($R^{2}$) and mean squared error (MSE) metrics were used to assess the accuracy of Kcat predictions.

The optimization process involves evolutionary operations beginning with a selection phase where the top-performing sequences, based on fitness scores, proceed to further evolution. During the crossover phase, amino acids from selected sequences are combined to produce new variants, introducing diversity and enabling the exploration of novel sequence spaces. We maintained a population size of 500 to preserve this diversity and limited our optimization to 30 generations, retaining the top 80% of sequences based on their fitness scores in each iteration.

### Statistical analysis of the fitness functions

In the comprehensive evaluation of enzyme mutants for enhanced catalytic performance, the study hinges on two pivotal metrics: the feasibility score ($F_{s}$) and the turnover number ($\text{Kcat}$). To quantify changes in enzyme functionality, we computed the difference in $F_{s}$ ($\Delta X_{reaction_{i}} = X_{mutant_{i}} - X_{WT_{i}}$ for $i = 0, 1, 2, \ldots , 105$). To statistically validate the observed changes, we employed the Mann–Whitney U rank test [34]. The same strategy has been applied to the $\text{Kcat}$.

### MD for structural validation

Starting with a selection of enhanced catalytic enzymes, specific mutations were introduced into the original protein structures from the PDB using the PyMOL mutagenesis tool [35]. These mutated structures were then refined and equilibrated using Gromacs [36], a comprehensive MD simulation suite, with the OPLS-AA/L all-atom force field [37] to accurately model interactions between proteins and solvents. Proteins were solvated in a cubic box with a minimum buffer of 1.0nm from the box edges, allowing for molecular movement within the simulation space.

A minimization phase was performed to remove steric clashes and energetically unfavorable conformations. This step utilized the steepest descent integrator with the maximum convergence criterion of 1000 kJ mol$^{-1}$nm$^{-1}$, a step size of 0.01 nm, and 50 000 steps. The equilibration process consisted of two phases: the NVT phase (constant number of particles, volume, and temperature) for 15 ns at 300 K, and NPT phase (constant number of particles, pressure, and temperature) for 50 ns at 300 K and 1atm. For more details on the simulation parameters, see Table S1.

To analyze the impact of mutations on protein behavior, the study quantified the phi ($\phi $) and psi ($\psi $) dihedral angles, crucial for understanding protein backbone conformation. These angles provide insight into the rotational freedom around the N-C$\alpha $ and C$\alpha $-C bonds, respectively, and are sensitive indicators of structural changes. By comparing these angles between wild-type (WT) and mutant proteins over the last 20 ns of each simulation, the structural impacts of mutations were discerned. MD simulation data were segmented into 100 ps snapshots for this analysis, allowing for a detailed temporal resolution of the conformational dynamics.

The mathematical function $f_{\text{sim}}(t^{\prime})$ was employed to quantify dynamic structural deviations in the protein over time. The function is defined as 


(1) \begin{align*}& f_{\text{sim}}(t^{\prime}) = \frac{\sum_{t=t^{\prime}}^{t_{\text{max}}} \left( \sum_{i=1}^{i_{\text{max}}} (\theta_{i}^{\text{wt}}(t^{\prime}) - \theta_{i}^{\text{sim}}(t))^{2} \right)}{(t_{\text{max}} - t^{\prime}) \cdot i_{\text{max}}}.\end{align*}

In Equation 1, $f_{\text{sim}}(t^{\prime})$ calculates the cumulative squared difference in dihedral angles $\theta $ at each amino acid position $i$ between the WT protein ($\theta _{i}^{\text{wt}}$) and the mutant protein ($\theta _{i}^{\text{sim}}$) across all considered positions up to $i_{\text{max}}$ for each time step $t^{\prime}$ within the simulation. By calculating $f_{\text{sim}}(t^{\prime})$ over the entire simulation trajectory, the study systematically evaluates how the introduced mutations impact the conformational dynamics of the protein backbone relative to the WT structure. Higher values of $f_{\text{sim}}(t^{\prime})$ indicate larger deviations in the dihedral angles and greater structural perturbations caused by the mutations. To further quantify the relationship between WT and mutant conformations, the Pearson correlation coefficients [38] were computed between their respective dihedral angle time series. This analysis reveals the extent to which the dihedral angle fluctuations of the mutants linearly correlate with those of the WT, providing insights into the effects of mutations on the protein’s backbone flexibility and dynamics. To assess the specificity and the impact of our best mutants, we created two types of control simulations: random mutations and proline mutations. For the random mutation controls, we created mutants by randomly mutating the same positions that were mutated in our best mutants. Importantly, we excluded the amino acids already present in those positions in the best mutants to ensure randomization. For each reaction, we generated five such mutants. This approach allowed us to compare the trajectories of our best mutants with a backdrop of random, nonspecific mutations. In the proline mutation controls, we introduced five consecutive prolines into a randomly chosen alpha-helix for each reaction. This strategy was employed to introduce significant structural changes, as proline residues are known to induce kinks and disrupt secondary structures, particularly alpha-helices [39, 40]. We generated one proline control mutant per reaction, providing a stark contrast to both our best mutants and the random mutation controls. We performed the same detailed analyses on these control mutants as on our best mutants, focusing on their deviations from the WT.

## Results

We present our results in three distinct parts. The first part evaluates various mutation strategies and their effects on the optimization process. The second part focuses on the optimization results using feasibility and catalytic efficiency (Kcat) as fitness functions. Finally, we offer an in-depth analysis of the MD of seven enzymatic reactions assessing the effect of mutations on their conformations.

### Protein language model for enzyme mutation

**Figure 2:**
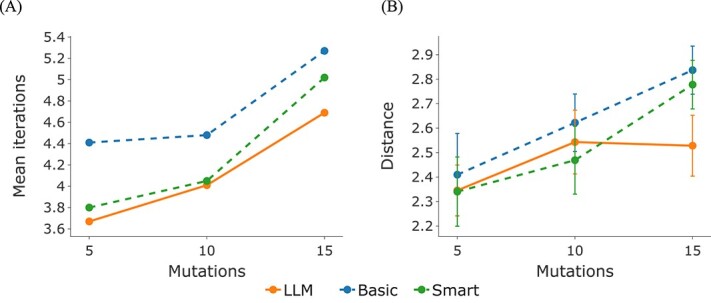
**Efficacy of mutation strategies in enzyme optimization**. (**A**) shows the average iterations to reach convergence for 27 enzymatic reactions using different mutation strategies. It highlights the LLM’s efficiency in suggesting beneficial mutations for faster optimal enzyme functionality. Meanwhile, the (**B**) graph compares the strategies by showing their Wasserstein distances from a theoretical reference distribution, representing the ideal case where algorithms converge in just one step. This comparison helps to quantify how closely each strategy approaches an ideal. The LLM strategy demonstrates lower distances, indicating superior performance, particularly with higher mutation counts.

A key element of our enzyme optimization is determining which amino acids to substitute in the protein sequence. Here, we evaluated three mutation strategies, one based on LLMs, and two leveraging transition matrices, which encode the probability of mutating one amino acid at a given position into another. As an LLM-based strategy, a model based on evolutionary scale modeling (ESM-2) [26] was considered, as it is a state-of-the-art language model tailored to protein sequences. Moreover, we considered two approaches leveraging transition matrices: (a) a transition matrix informed by conservation analysis of homologous sequences (named “Smart”), derived through Psi-Blast [28] and multiple sequence alignments (MSA) [41], which emphasizes mutations conserved across species, highlighting their biological relevance; and (b) a baseline transition matrix (named “Basic”), which assigns equal probability to all amino acid replacements, serving as a control. For each of these approaches, we assessed the effect of the respective mutations on feasibility when 5, 10, and 15 mutations were allowed. We examined the optimization of 27 distinct reactions. The results demonstrated that the LLM strategy achieves faster convergence, as evidenced by reaching a plateau in the optimization score with fewer iterations compared with other methods (see Fig. 2A).

To quantify the convergence speed of the different strategies, we employed the Wasserstein distance metric [29] (for more details, see Materials and Methods). This analysis revealed that as the maximum number of allowed mutations per sequence increased, the LLM strategy exhibited a significantly steeper decline in Wasserstein distance compared with both the Smart and Basic transition matrices (see Fig. 2B). This indicates that the LLM-based mutation strategy exhibits superior performance, particularly in scenarios that permit a higher degree of sequence variations. This analysis underscores the LLM strategy’s potential to outperform more traditional mutation approaches, especially under conditions that allow for extensive sequence modification.

Statistically significant differences ($P$-value $<0.05$, Wilcoxon test [42]) were observed between the LLM strategy and transition-matrix-based strategies in terms of Wasserstein distance, particularly for larger mutation counts (maximum of 15 mutation). These results highlight the efficiency of the LLM strategy in suggesting amino acid replacements when compared with probability-based approaches like transition matrices. This efficiency translates to its superior ability to explore the vast possibilities within the combinatorial optimization space.

### Optimizing enzyme feasibility and turnover dynamics

Another component of our methodology is evaluating of the generated mutants. We carried out this assessment through two distinct fitness functions: (a) feasibility score, which reflects the probability of a specific enzyme mutant to catalyze a given reaction (referred to as the *feasibility score* or $F_{s}$) and (b) the predicted catalytic efficiency ($\text{Kcat}$), which captures the efficiency in converting substrate molecules into a product per unit time.

**Figure 3:**
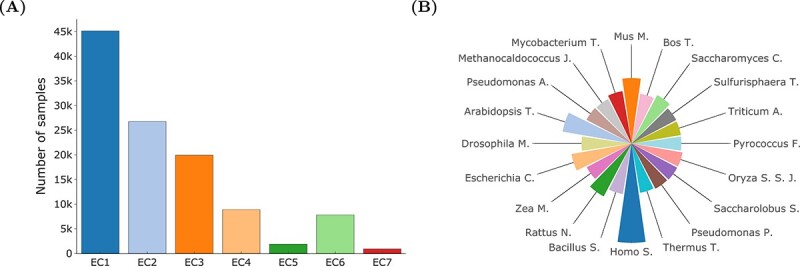
**Overview of dataset composition**. (**A**) Shows the distribution of reactions within the training set by EC numbers, illustrating the diversity of enzyme categories. Figure (**B**) highlights the 20 most abundant organisms, showcasing the broad range of biological entities encompassed in our dataset.

Both fitness functions were based on machine learning models, trained on biocatalysed reactions sourced from BRENDA database [21]. Models were trained to distinguish “reported” reactions in literature from negative samples, created by randomly combining substrates, products, and enzymes (for more details, see Materials and Methods). To ensure broad applicability, our dataset includes reactions from all EC classes (see Fig. 3(A)) and features 2562 distinct organisms (see Fig. 3(B)), thereby guaranteeing a comprehensive representation of biochemical diversity.

**Figure 4:**
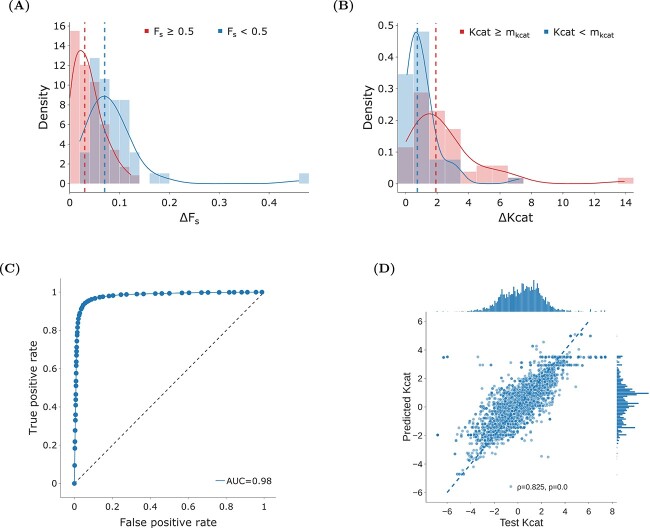
**Assessing feasibility and turnover in enzyme optimization**. (**A**) compares $\Delta $$F_{s}$, which represents the change in feasibility scores, among mutants with initial low feasibility analysis (blue) and high feasibility (red). Notably, the denser peak for the blue curve suggests significant improvement in feasibility for mutants with lower initial scores. (**B**) shows $\Delta $$\text{Kcat}$ changes of enzymes, split around the median ($m_{\text{Kcat}}$). Improvement is observed in both low and high initial $F_{s}$ reaction categories. Graph (**C**) illustrates our RF model’s accuracy in determining biocatalytic reaction feasibility, achieving an AUC of 0.986. The scatter plot (**D**) compares predicted and observed $\text{K}_{\text{cat}}$ values, indicating a strong positive relationship with statistical significance. Histograms highlight dataset central tendency and dispersion.

The feasibility metric is critical for excluding mutants that are unlikely to improve the specific biocatalytic reaction of interest, ensuring only those with a real potential for enhancing the process are considered. An RF model [30] was trained to predict feasibility using learned embeddings for substrates, products [31], and enzymes [26] as input (see details in Materials and Methods). This model achieved an area under the curve (AUC) of 0.98 on the test set showing its accuracy in predicting reaction feasibility (see Fig. 4(C)). We employed the $F_{s}$ metric to guide the iterations in our optimization process, which led to improvements in the predicted feasibility score of 95 out of 105 reactions (90 %)in our test set. This demonstrates the GA’s effectiveness in guiding the optimization toward more beneficial biocatalytic improvements. Among the optimized reactions, eight had $F_{s}$ values akin to “random” associations ($F_{s} < 0.5$) and were optimized to attain final $F_{s}$ values comparable with “reported” associations. This showcases our algorithm’s ability to significantly improve the feasibility of certain reactions with no more than five mutations, making them comparable with known, effective biochemical reactions.

Further investigation highlighted a distinct pattern: reactions with initial low feasibility ($F_{s} < 0.5$) underwent larger increases in optimization scores than those with initially higher feasibility ($F_{s} \geq 0.5$), as illustrated in Fig. 4(A). This indicates that reactions that are already optimal (in terms of $F_{s}$) pose a greater challenge for further enhancement. Conversely, reactions derived from random associations displayed greater potential for optimization. This finding suggests the potential to transform seemingly arbitrary reactions into viable biological processes with minimal mutations. Statistical analysis, conducted using a Mann–Whitney U rank test [34] confirmed a significant difference ($P$-value $<0.05$) in the optimization outcomes between these two groups of reactions, underscoring the effectiveness of our strategy in improving reaction feasibility.

For the second fitness function, we employed an XGBoost [32] model that uses enzyme and substrate information to predict the corresponding $\text{Kcat}$ values. The model exhibits high predictive accuracy, as indicated by an MSE of 0.93, an R-squared value of 0.68, and a Pearson correlation coefficient of 0.83 (see Fig. 4(D)). These metrics demonstrate the model’s capability to accurately capture the complexities of catalytic efficiency, showcasing performance comparable with state-of-the-art models [43].

When employing $\text{Kcat}$ as the guiding fitness function within our optimization framework, we optimized the entire test set. Segmenting our test set into two subsets based on the median $\text{Kcat}$ (denoted $m_{\text{Kcat}}$) enabled a comparative analysis between reactions with initially low $\text{Kcat}$ ($\text{Kcat} < m_{\text{Kcat}}$) and those with high initial values ($\text{Kcat} \geq m_{\text{Kcat}}$). We noted that reactions with lower initial $\text{Kcat}$ values showed, on average, smaller improvements in $\Delta \text{Kcat}$ than reactions with higher initial values. This discrepancy in optimization was statistically significant ($P$-value $<0.05$) based on the Mann–Whitney U rank test. This indicates a distinct pattern in the distribution of $\Delta \text{Kcat}$ improvements between the two subsets. It suggests that reactions starting with higher catalytic efficiency might have a higher potential for optimization. This phenomenon could be attributed to an already favorable starting point in terms of sequence for catalyzing specific reactions, making them more amenable to minimal mutational changes for enhancing catalytic performance within a specified exploration time frame.

### Prospective enzyme optimization and in silico validation

We evaluated the enzyme mutants generated by our optimization framework using MD simulations. These simulations focused on seven enzymatic reactions that demonstrated significant improvements in feasibility scores, transitioning from low to high feasibility post-optimization (see Figure S1). The optimization focused on enhancing the $F_{s}$ metric as the primary fitness function, rather than $\text{Kcat}$, to assess the potential improvement in enzyme performance. The analyzed reactions showcased a wide range of enzyme functionalities, from amino transferases (see Figure S1A) to isomerases (see Figure S1B), highlighting the broad applicability of our optimization approach. We analyzed the mutants by conducting simulations under two ensembles: (a) NVT ensemble and (b) NPT ensemble. Upon equilibration, we monitored the phi ($\phi $) and psi ($\psi $) dihedral angles of each amino acid residue for the last 20 ns of the equilibration.

The deviation of the dihedral angles between mutants and WT revealed a complex landscape of conformational dynamics across time and various amino acid positions. Both the WT enzymes and the mutants maintained a relatively stable trajectory of dihedral angles, although with a diverse range of deviations. These deviations, denoted as $f_{\text{sim}}$, were measured by calculating the squared differences from the WT dihedral angles. Despite the observed variability among mutants, no consistent trends in conformational changes significantly distinguished the mutants from the WT across all examined reactions (see Figure S2 and Figure S3). Based on dihedral angle metrics, our findings suggest that the mutations may not lead to widespread or significant structural alterations in the proteins.

**Figure 5:**
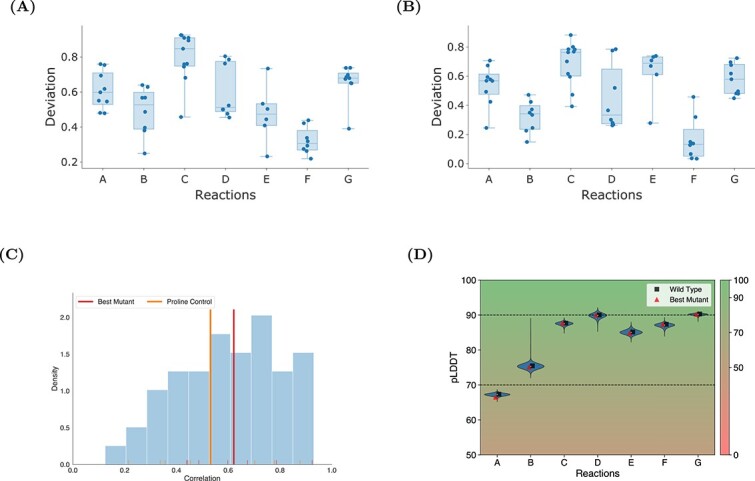
**Foldability and Pearson correlation analysis of dihedral angle deviations in optimized enzymes**. (**A**) Deviation of correlations between $\phi $ angles of mutants and respective WT. (**B**) Deviation of $\psi $ angles. (**C**) S distribution of correlation coefficients between deviations of control mutations, best mutants, and their wild types. The red line represents best mutants, and the orange line represents proline control mutants, showing proline controls exhibit lower correlations to the WT. All observations are statistically significant with $P$-value $<0.05$. (**D**) PLDDT plot of all generated sequences. WT-type values (black squares) and best mutant values (red triangles) are shown against a pLDDT scale background, with dashed lines indicating confidence thresholds (50–70: low, 70–90: confident, $>90$: high).

We further assessed the variation in the $\phi $ and $\psi $ angles by examining the Pearson correlation coefficients between the WT and each mutant. This analysis, depicted in Fig. 5(A), reveals that the $\phi $ angle correlations generally show a moderate positive correlation across the reactions (see Fig. 5(A)). These correlations are significant ($P$-value $<0.05$) indicating that the mutants primarily induce conformational shifts in $\phi $ angles that remain largely aligned with the WT behavior. In contrast, the $\psi $ angle correlations, shown in Fig. 5(B), present a more heterogeneous picture. Reaction F displays a narrower spread in correlation coefficients, with medians lower than those observed for $\phi $ angles. This suggests a potential difference in how these dihedral angles respond to mutations. $\psi $ angles appears to be more responsive to the structural perturbations caused by mutations. Despite these variations, it is crucial to note that the absence of a uniform trend in conformational changes persists. While some individual mutants occasionally show very low deviations, indicating temporary structural shifts, these anomalies do not translate in a persistent pattern over time. Therefore, the mutational impact, as inferred from our correlation analysis, does not point to substantial or pervasive structural modifications within the protein’s architecture. This reinforces our initial findings that the examined mutations, although capable of inducing localized and temporary changes, may not have a significant long-term impact on the protein’s structure. We performed two additional types of control simulations: random mutations and proline mutations. Our results revealed that random mutation controls on average behaved similarly to our best mutants. Proline mutation controls, on the other hand, exhibited trajectories that varied from the WT. The introduction of consecutive prolines led to marked deviations, highlighting the profound impact of such structural perturbations on protein dynamics.

### Foldability analysis of the mutated proteins

The foldability of each sequence was assessed by computing the predicted Local Distance Difference Test (pLDDT) scores using the ESM-Fold algorithm [26]. ESM-Fold is a state-of-the-art protein structure prediction tool that provides high-accuracy models of protein folding, allowing us to rigorously evaluate the structural integrity of our sequences. The analysis revealed that the foldability of the mutated sequences was comparable with that of the WT. The pLDDT scores, which range from 0 to 100, indicate the confidence of the predicted structures. Higher scores suggest more reliable and stable protein folds. As illustrated in Fig. 5(D), where the black squares represent the pLDDT values of the WT and the red triangles represent those of the best mutants, the distribution of pLDDT scores for our mutated sequences closely matches that of the WT sequences. This similarity in foldability scores demonstrates that the mutations introduced in our study do not significantly compromise the structural integrity of the proteins.

## Discussion

This study presents a novel enzymatic optimization framework that leverages the combined strengths of LLMs and GAs. Our methodology efficiently identified and implemented mutations to enhance predicted protein functionality. This approach facilitates a systematic and iterative optimization of enzyme sequences. This results in variants with significantly improved feasibility scores and predicted turnover rates ($\text{Kcat}$) across a spectrum of biocatalytic reactions. Our comprehensive evaluation on a dataset of 105 biocatalytic reactions demonstrates the effectiveness of this approach in improving feasibility scores and turnover.

The validity of our optimization pipeline was further corroborated by MD simulations. These simulations confirmed the structural stability of the enzymes optimized with our methodology. These simulations highlight our approach’s ability to generate mutated sequences that maintain dynamics similar to their WT while having a higher likelihood of catalyzing the targeted reaction. We acknowledge the inherent limitations of our framework as a heuristic method. However, while it may not always guarantee the absolute best solution, it consistently yields high-quality outcomes.

The integration of LLMs and GAs into enzyme optimization enables precise control over enzyme modification sites. This advances the development of novel biocatalysts and enhances the performance of existing enzymes. Our framework is designed to be highly modular, enabling the integration of various metrics for enzyme performance. Herein, we showcased an application focusing on modeling feasibility and $\text{Kcat}$. Numerous other attributes crucial for enzymes function, such as thermostability [44, 45], pH tolerance [46], and foldability [47], can be incorporated into the fitness score, enabling a comprehensive optimization.

In conclusion, this study showcases the power of the LLM–GA hybrid methodology in enzyme optimization, paving the way for the computational design of biocatalysts, which hold the potential to contribute to more efficient and sustainable chemical processes.

**Key Points**

- This study introduces a framework that combines the predictive power of large language models with the adaptive mechanisms of genetic algorithms to accelerate enzyme design.
- The proposed framework has been applied to a dataset of 105 biocatalytic reactions, focusing on improving the feasibility of the reactions and increasing the enzymes’ turnover rate (Kcat).
- The approach achieved a 90% improvement in predicted feasibility across the test set and a 100% improvement in Kcat values compared with the wild-type enzymes.
- Molecular dynamics simulations confirmed the structural stability of the optimized enzymes, demonstrating the ability of our framework to generate mutated sequences that maintain dynamics similar to the wild-type while improving catalytic performance.

## Supplementary Material

Enzeptional_Supplementary_Materials_Clean_bbae675

## Data Availability

The source code for this study is available via the GT4SD library [48] and can be accessed via this url: https://github.com/GT4SD/gt4sd-core/tree/main/examples/enzeptional.
