# Supplementary material for: Integrating genetic algorithms and language models for enhanced enzyme design
Source: Brief Bioinform. 2025 Jan 8;26(1):bbae675. doi: 10.1093/bib/bbae675 (PMC11711099; doi:10.1093/bib/bbae675)
Supplement: Enzeptional_Supplementary_Materials_Clean_bbae675 [file enzeptional_supplementary_materials_clean_bbae675.pdf]

Integrating Genetic Algorithms and Language Models for Enhanced Enzyme Design

Yves Gaetan Nana Teukam[1,2,*], Federico Zipoli[1,3], Teodoro Laino[1,3], Emanuele Criscuolo[2], Francesca Grisoni[2,4], and Matteo Manica[1]

[1]*IBM Research Europe, CH-8803 Rüschlikon, Switzerland*
[2]*Institute for Complex Molecular Systems and Dept. Biomedical Engineering, Eindhoven University of Technology, Netherlands*
[3]*National Center for Competence in Research-Catalysis (NCCR-Catalysis), Switzerland*
[4]*Centre for Living Technologies, Alliance TU/e, WUR, UU, UMC Utrecht, Netherlands*
[*]*Corresponding author: yna@zurich.ibm.com*

June 21, 2024

Supplementary Materials

Assumptions and limitations of the Wilcoxon and Mann-Whitney tests. macro:

macro:

The Wilcoxon test is a non-parametric statistical method suitable for analyzing paired data that need not follow a normal distribution. This test is particularly useful when the data distribution is skewed or unknown, as it does not make assumptions about normality. In our study, we employed the Wilcoxon test to compare Wasserstein distances between different mutation strategies in a combinatorial optimization problem. While the Wilcoxon test is robust to non-normal distributions, its statistical power is depends on sample size. Larger sample sizes generally increase the test's ability to detect significant differences between groups. To ensure sufficient statistical power in our analysis, we utilized 30 random seeds, effectively generating 30 independent samples for each mutation strategy. Conversely, the Mann-Whitney U test compares two independent groups across a continuous or ordinal dependent variable. This test is relevant for assessing variables such as ΔF_s and ΔKcat, where groups are independent and the variables are continuous in nature, satisfying the test's requirements. However, its ability to identify significant differences between groups can be influenced by sample size. While larger samples facilitate the detection of minor differences, the Mann-Whitney U test may not offer the same sensitivity to effect size variations as parametric tests.

Addressing overfitting in feasibility and Kcat prediction models. To prevent overfitting in our feasibility and Kcat prediction models, we implemented several robust strategies. Both models utilized 5-fold cross-validation, where the dataset was split into five parts. The model was trained on four parts and validated on the remaining part, with this process rotated through all parts. The 5-fold cross-validation results were comparable to those of the wild type, demonstrating the robustness of our approach. The Random Forest (RF) model for feasibility prediction comprised 100 decision trees, each trained on a bootstrap sample of the data using random feature subsets. The Gini impurity criterion selected optimal splits at each node, preventing overly complex models and overfitting. The average cross-validation score for the RF model was 0.98. For the Kcat prediction model using the XGBoost algorithm, several strategies were employed: (i) hyperparameters were carefully tuned to balance model complexity and performance. The settings included a learning rate of 0.09, max delta step of 1.19, min child weight of 2.82, reg alpha of 1.94, and reg lambda of 4.95;

(ii) A logarithmic transformation was applied to the dataset prior to training to improve linearity and reduce the impact of outliers. (iii) 5-fold cross-validation was performed, with the average cross-validation Mean Squared Error (MSE) being 0.98.

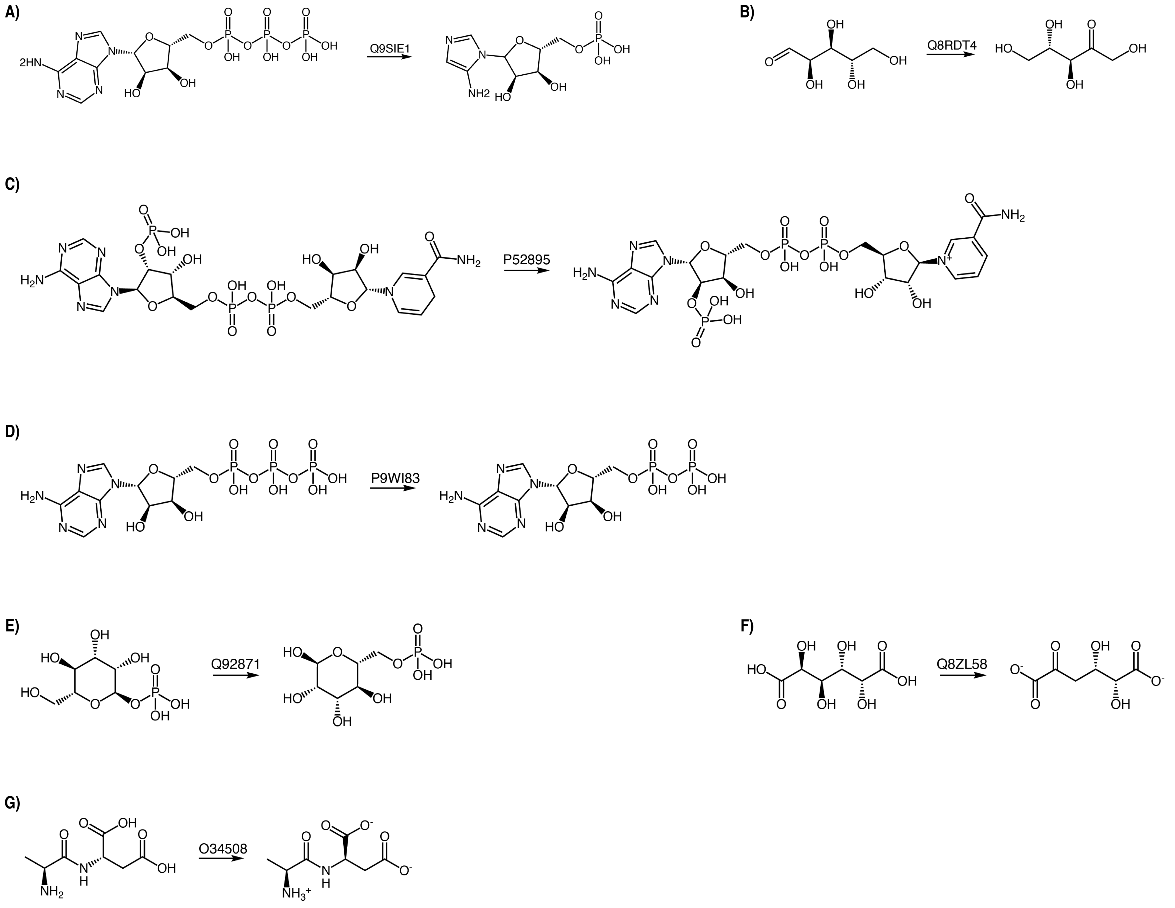

Figure S1: **Seven reactions selected for in silico validation.** Each reaction is accompanied by the UniProt Accession Number of the randomly selected protein chosen for optimization. Reaction A is known to be catalyzed by Phosphoribosylformylglycinamidine cyclo-ligases, like P74883 and P52424. In our optmization, we started from an aminotransferase (Q9SIE1). Reaction B, in contrast, involves the catalytic activity of isomerases; we started from an L-methionine gamma-lyase (Q8RDT4). Reactions C and D are typically catalyzed by a broad spectrum of over 20 different enzyme types. For our study, we randomly employed a reductase and a kinase to catalyze reactions C and D, respectively. Reactions E, F, and G are catalyzed by phosphomannomutase, dehydratase, and epimerase, respectively.

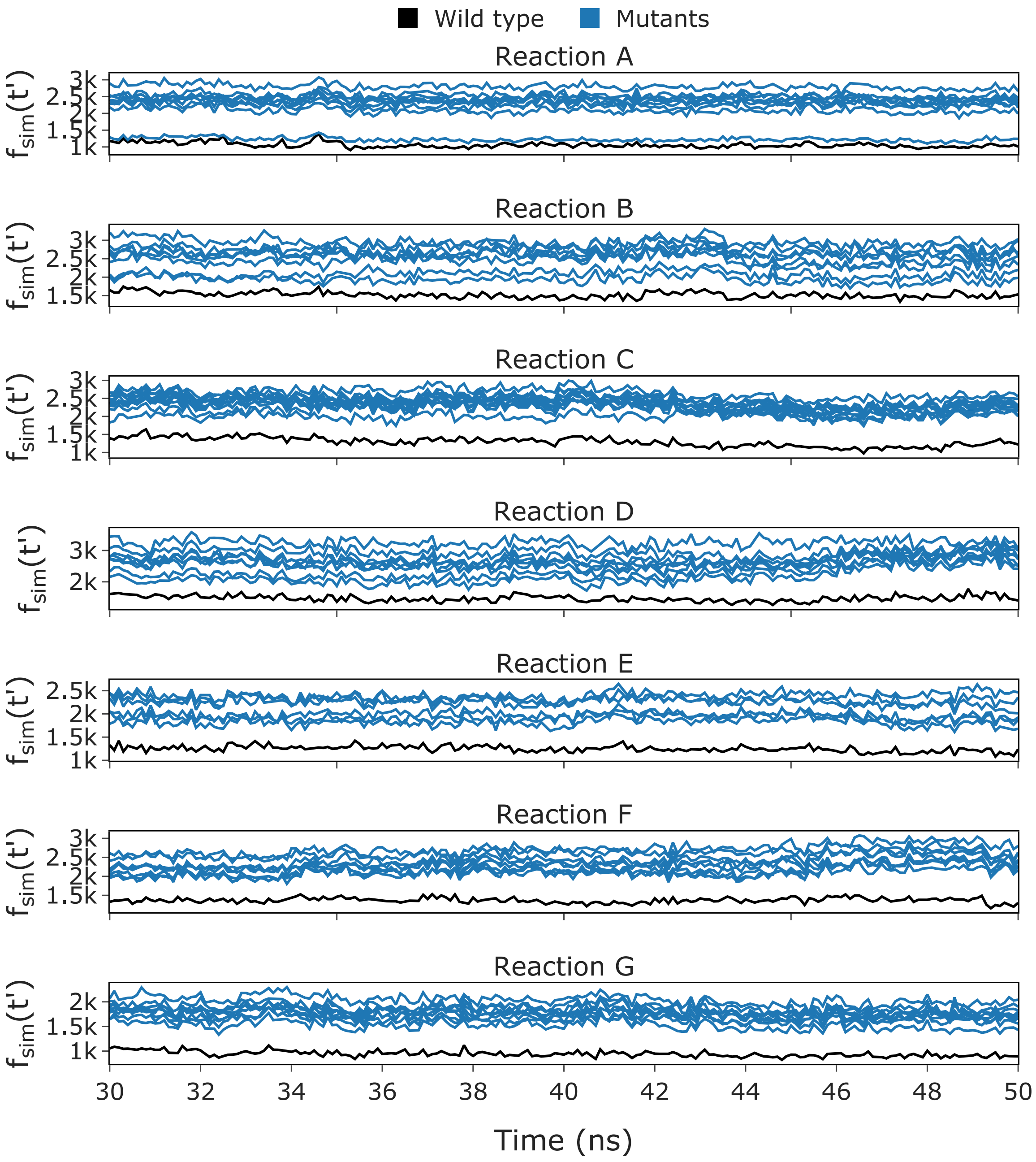

Figure S2: Φ **angle fluctuations.** Trajectories of squared distances in the ϕ dihedral angles over time for seven different enzymatic reactions (A-G) during molecular dynamics simulations. Wild type enzymes are indicated by black lines, while the blue lines represent the array of mutants for each reaction. These data illustrate the dynamic variability in the ϕ angles among the mutants compared to the relative stability observed in the wild-type enzymes, without showing a uniform pattern of conformational change across the analyzed reactions.

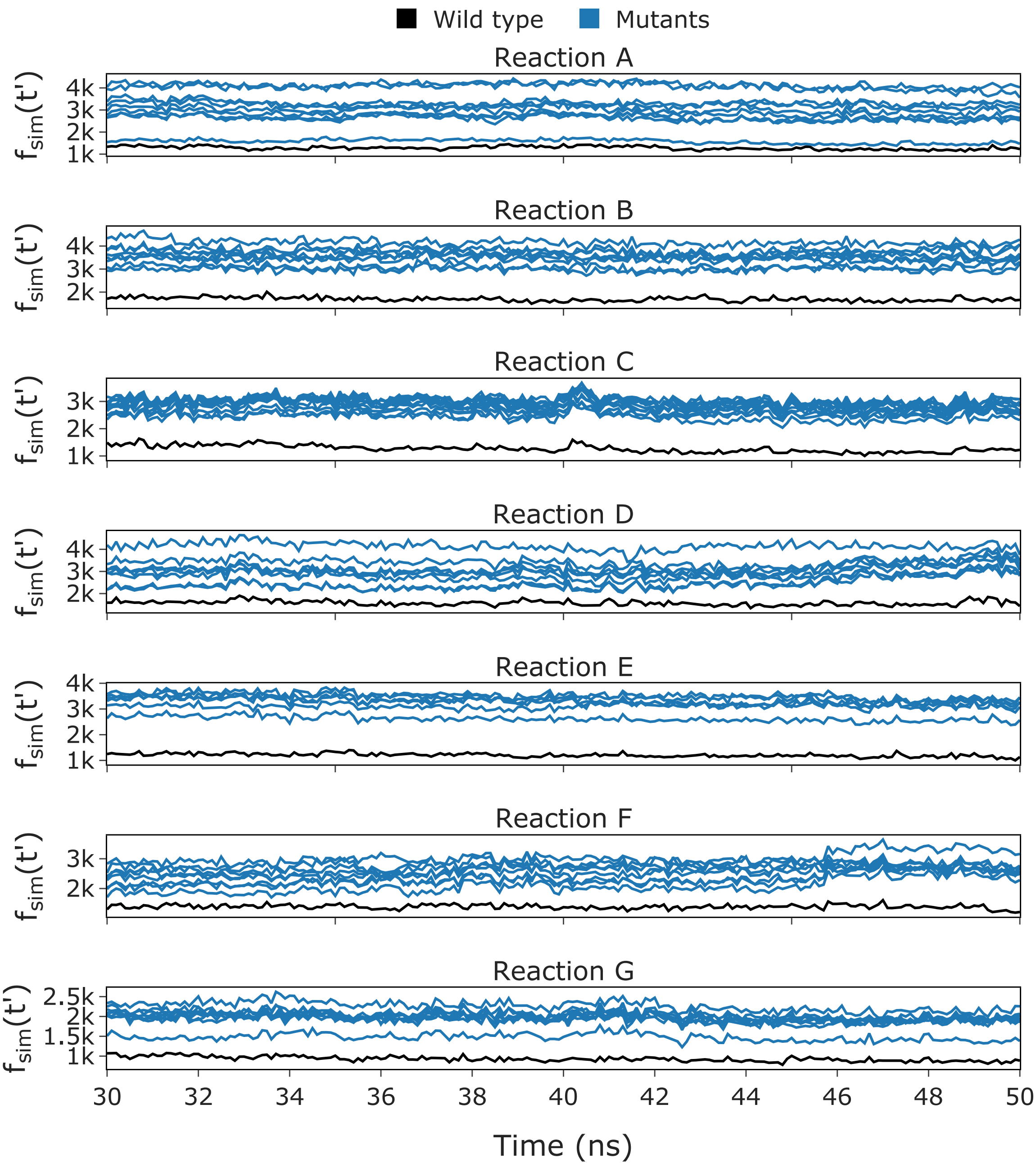

Figure S3: **Ψ angle fluctuations.** Plot depicting the squared fluctuations in the ψ dihedral angles over the molecular dynamics simulation for seven distinct enzymatic reactions (A-G). The black lines represent the wild type enzymes, maintaining a consistent trajectory, whereas the blue lines indicate the mutants' trajectories, which exhibit varied deviations from the wild type, reflecting the conformational diversity introduced by mutations.

	Parameter Name	Description	Value
Minimization	integrator	Algorithm used	steep
	emtol	Maximum convergence criterion (kJ/mol)	1000.0
	emstep	Energy step size (nm)	0.01
	nsteps	Maximum number of minimization steps	50000
	coulombtype	Treatment of long-range electrostatic interactions	PME
	rcoulomb	Long-range electrostatic cut-off (nm)	1.2
	rvdw	Long-range Van der Waals cut-off (nm)	1.2
	pbc	Periodic boundary conditions	xyz
NVT	integrator	Algorithm used	md
	dt	Time step (ps)	0.002
	nsteps	Maximum number of steps	7500000
	Tcoupl	Temperature coupling algorithm	V-rescale
	tc_grps	Temperature coupling groups	Protein Non-Protein
	tau_t	Time constant for temperature coupling (ps)	0.1 0.1
	ref_t	Reference temperature (K)	300 300
NPT	integrator	Algorithm used	md
	dt	Time step (ps)	0.002
	nsteps	Maximum number of steps	25000000
	Tcoupl	Temperature coupling algorithm	V-rescale
	tc_grps	Temperature coupling groups	Protein Non-Protein
	tau_t	Time constant for temperature coupling (ps)	0.1 0.1
	ref_t	Reference temperature (K)	300 300
	Pcoupl	Pressure coupling algorithm	C-rescale
	Pcoupltype	Pressure coupling type	isotropic
	tau_p	Time constant for pressure coupling (ps)	1.0
	ref_p	Reference pressure (bar)	1.0

Table S1: Simulation parameters
